# Supplementary material for: Effects of Sex on the Susceptibility for Atrial Fibrillation in Pigs with Ischemic Heart Failure
Source: Cells. 2023 Mar 23;12(7):973. doi: 10.3390/cells12070973 (PMC10093477; doi:10.3390/cells12070973)
Supplement: Supplementary file 1 [file cells-12-00973-s001.zip › cells-2274886-supplementary.pdf]

Effects of Sex on the Susceptibility for Atrial Fibrillation in Pigs with Ischemic Heart Failure

Valerie Pauly [1,2,3], Julia Vlcek [1,3], Zhihao Zhang [1,2,3], Nora Hesse [1,2,3], Ruibing Xia [1,2,3], Julia Bauer [1,2,3], Simone Loy [1,2,3], Sarah Schneider [1,2,3], Simone Renner [4,5,6,7], Eckhard Wolf [4,5,6,7,8], Stefan Kääb [1,2,4], Dominik Schüttler [1,2,3], Philipp Tomsits [1,2,3,†] and Sebastian Clauss [1,2,3,4,*,†]

[1] Grosshadern Campus, Department of Medicine I, University Hospital Munich, Ludwig-Maximilians-University (LMU), Marchioninistrasse 15, Munich, D-81377, Germany
[2] German Center for Cardiovascular Research (DZHK), Partner Site Munich, Munich Heart Alliance, Munich, D-81377, Germany
[3] Institute of Surgical Research at the Walter-Brendel-Centre of Experimental Medicine, University Hospital Munich, LMU Munich, Marchioninistrasse 68, Munich, D-81377, Germany
[4] Interfaculty Center for Endocrine and Cardiovascular Disease Network Modelling and Clinical Transfer (ICONLMU), LMU Munich, Feodor-Lynen-Strasse 19,Munich, D-81377, Germany
[5] Chair for Molecular Animal Breeding and Biotechnology, Gene Center and Department of Veterinary Sciences, LMU Munich, Feodor-Lynen-Strasse 25, Munich, D-81377, Germany
[6] Center for Innovative Medical Models (CiMM), Department of Veterinary Sciences, LMU Munich, Hackerstrasse 27, Oberschleissheim, D-85764, Germany
[7] German Center for Diabetes Research (DZD), Ingolstädter Landstrasse 1, Neuherberg, D-85764, Germany
[8] Laboratory for Functional Genome Analysis (LAFUGA), Gene Center, Grosshadern Campus, LMU Munich, Feodor-Lynen-Stasse 25, Munich, D-81377, Germany
* Correspondence: sebastian.clauss@med.uni-muenchen.de
† These authors contributed equally to this work.

Item	Page
Supplemental Figure S1: Hemodynamic Parameters	2
Supplemental Figure S2: Sinus Node Recovery Time (SNRT)	3
Supplemental Figure S3: Corrected Sinus Node Recovery Time (SNRT/BCL) in animals with and without Atrial Fibrillation (AF)	4

Supplemental Figure S1: Hemodynamic Parameters

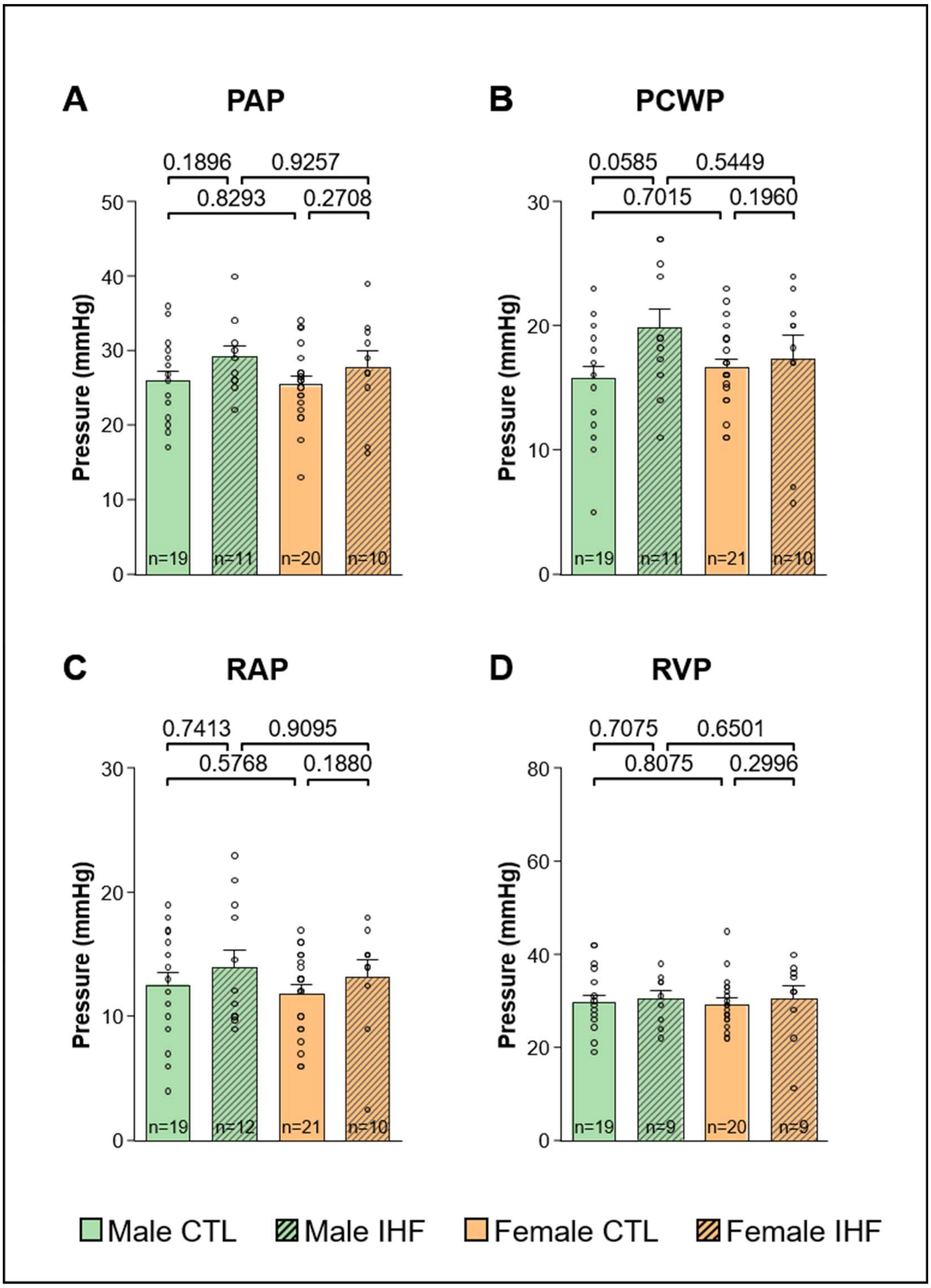

Supplementary Figure S1. Hemodynamic Measurements A, Systolic pulmonary arterial pressure (PAP). **B,** Systolic pulmonary capillary wedge pressure (PCWP). **C,** Systolic right atrial pressure (RAP). **D,** Systolic right ventricular pressure (RVP). *CTL*, control animals without ischemic heart failure; *IHF*, animals with ischemic heart failure. Graphs shown as MEAN+SEM, grey circles represent data of individual pigs. Mann-Whitney-U Test.

Supplemental Figure S2: Sinus Node Recovery Time (SNRT)

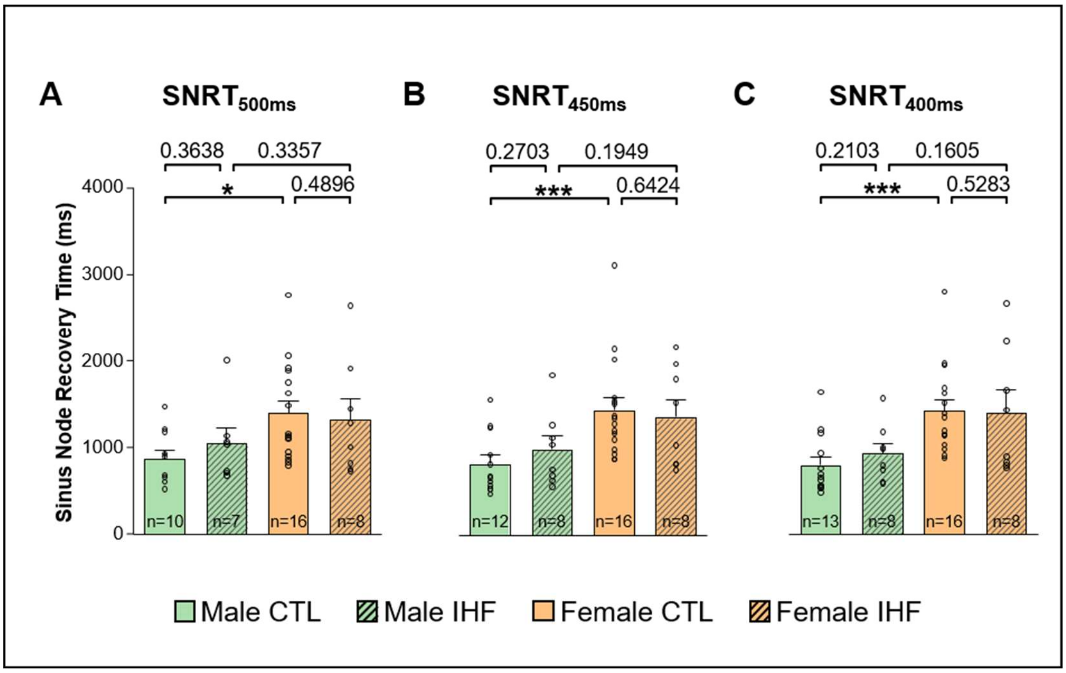

Supplementary Figure S2. Sinus Node Recovery Time (SNRT). A, SNRT at a pacing cycle length of 500 ms. **B,** SNRT at a pacing cycle length of 450 ms. **C,** SNRT at a pacing cycle length of 400 ms. *CTL*, control animals without ischemic heart failure; *IHF*, animals with ischemic heart failure. Bar graphs represent Mean+SEM, grey circles represent data of individual pigs. Mann-Whitney-U Test. *p<0.05; ***p<0.001**.**

Supplemental Figure S3: Corrected Sinus Node Recovery Time (SNRT/BCL) in animals with and without Atrial Fibrillation (AF)

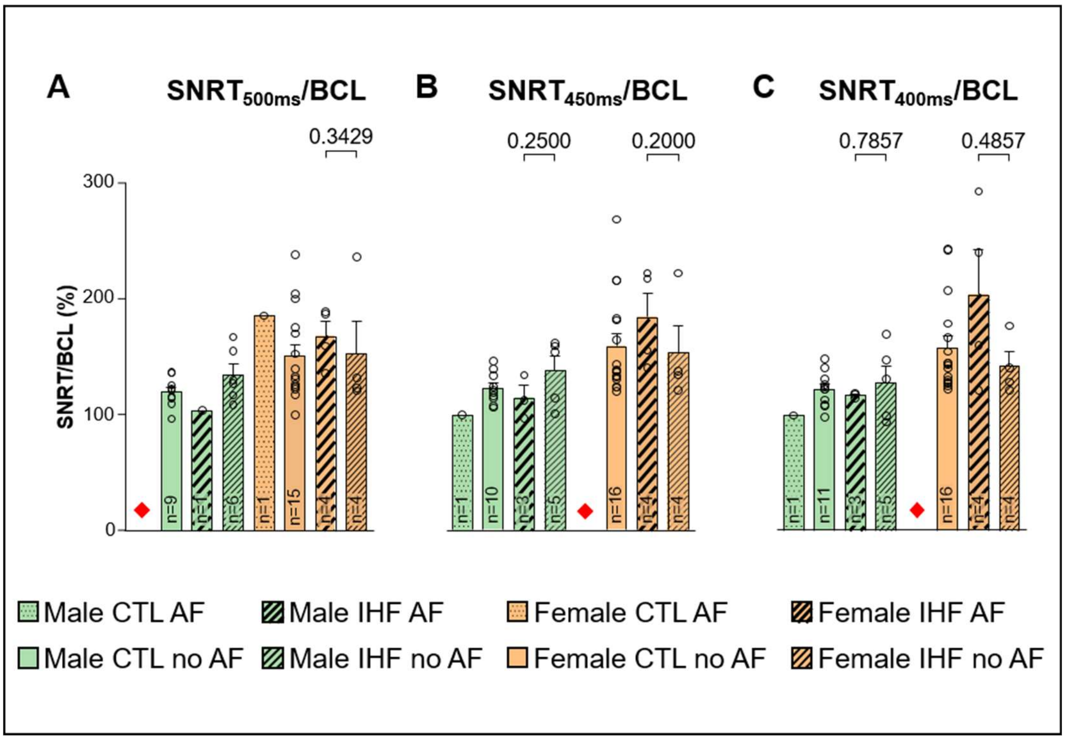

Supplementary Figure S3. **Corrected Sinus Node Recovery Time (SNRT/BCL) in animals with and without Atrial Fibrillation (AF). A,** SNRT/BCL at a pacing cycle length of 500 ms. **B,** SNRT/BCL at a pacing cycle length of 450 ms. **C,** SNRT/BCL at a pacing cycle length of 400 ms. *CTL*, control animals without ischemic heart failure; *IHF*, animals with ischemic heart failure. Bar graphs represent Mean+SEM, grey circles represent data of individual pigs. In some subgroups no AF episode was observed, these groups are marked with red diamonds (n=0). Mann-Whitney-U Test where applicable.
